# Authors’ Reply

**DOI:** 10.18502/jovr.v17i2.10825

**Published:** 2022-04-29

**Authors:** Navid Manafi, Kaveh Abri Aghdam

**Affiliations:** ^1^Department of Ophthalmology, David Geffen School of Medicine, University of California-Los Angeles, Los Angeles, CA, United States; ^2^Eye Research Center, Eye Department, The Five Senses Health Institute, School of Medicine, Iran University of Medical Sciences, Tehran, Iran

##  Dear Editor,

We would like to thank Dr Siddharth Madan and colleagues for their interest in our work.^[1]^ We found that the majority of temporal artery biopsies (TABs) led to negative results and giant cell arteritis (GCA) could be diagnosed based on clinical grounds rather than relying just on TAB.

The American College of Rheumatology (ACR) formulated its classification criteria for diagnosing GCA in 1990.^[2]^ These criteria were used for the classification of and not for early diagnosis of GCA. The revised ACR criteria were proposed as a diagnostic tool for earlier diagnosis of GCA in 2016.^[3]^

TAB has been considered the gold standard test for the diagnosis of GCA but it has suboptimal sensitivity and specificity. One of the main limitations of TAB is the presence of “skip lesions”, which increases the false-negative rate. Previous studies have revealed that an increase in TAB lengths or cut sections of the specimen does not yield a higher true positive rate.^[1,3,4]^

Some imaging modalities have been suggested as surrogates for TAB.^[5,6,7]^ However, they are not currently included in the ACR or other guidelines for diagnosing GCA.[2, 8] Currently, these modalities are compared with the TAB as a gold standard test, which is an imperfect standard itself. Color Doppler Ultrasound (CDUS) and high-resolution magnetic resonance imaging (MRI) with MR angiography (MRA) have been studied regarding their role in diagnosing GCA.^[7,9]^ The heterogeneous conclusions about the utility of CDUS likely reflect the operator-dependent nature of the procedure and may also result from the variability of the clinical context, probe settings, technique, and equipment. Standardization of these factors may lead to more widespread use of CDUS for the diagnosis of GCA. MRA revealed to have a pooled sensitivity and specificity of 93% and 81%, respectively, when TAB was used as the reference standard.^[10]^

Fluorescein angiography (FA) is an invasive test that shows delayed choroidal filling and/or retinal artery in 56% of patients with arteritic anterior ischemic optic neuropathy (AAION) and even in some cases of GCA without visual symptoms but not in non-arteritic anterior ischemic optic neuropathy (NAION).^[11]^ Optical coherence tomography angiography (OCTA) non-invasively images capillary perfusion at various levels of the retina and optic disc. It could show dilation and eventual attenuation of the superficial peripapillary capillaries in eyes with AAION, corresponding with visual field loss and might be used as an adjunctive imaging modality.^[6,12,13]^ However, OCTA alone cannot differentiate NAION from AAION.

The increasing use of imaging modalities as diagnostic or adjunctive complements to TAB is promising. The use of TAB cannot be overlooked as it shows the actual pathology of the specimen and other modalities have not shown superior diagnostic values compared with TAB. However, guidelines and criteria need to be updated and include modern imaging technologies. Imaging modalities can also aid in the evaluation of other extracranial and intracranial arteries that might be affected by either GCA or other vasculitides.
